# Beauty standards and reproductive health: increasing use of GLP-1 receptor agonists despite limited evidence

**DOI:** 10.3389/fendo.2026.1911235

**Published:** 2026-09-15

**Authors:** Sophia Martins Simon de Matos, Lilith Schuler Faccini, Fernanda Sales Luiz Vianna, Lucas Rosa Fraga

**Affiliations:** 1 Graduate Program in Medicine, Medical Sciences, School of Medicine, Federal University of Rio Grande do Sul (UFRGS), Porto Alegre, Rio Grande do Sul, Brazil; 2 Experimental Research Center, Hospital de Clínicas de Porto Alegre (HCPA), Porto Alegre, Rio Grande do Sul, Brazil; 3 Teratology Information System (SIAT), Medical Genetics Service, Hospital de Clínicas de Porto Alegre (HCPA), Porto Alegre, Rio Grande do Sul, Brazil; 4 Department of Morphological Science, Institute of Health Sciences, Federal University of Rio Grande do Sul (UFRGS), Porto Alegre, Rio Grande do Sul, Brazil

**Keywords:** GLP-1 receptor agonist, obesity, periconceptional exposure, pregnancy, reproductive decision making, semaglutide

## Introduction

In ancient societies, when humans were still organized into nomadic societies and relied on hunting and gathering for food, the aesthetic standard for the female body was quite different from today. The Venus of Willendorf is a representation of what was beautiful and desired for women 28, 000 years ago: a large, curvy body. This body is directly related to the importance of abundance and its relationship to fertility in prehistoric societies (1). Nowadays, a different reality is observed, with humans spending more time sedentary and eating more unhealthy foods (2, 3). This is directly associated with increased Body Mass Index (BMI) and the increased prevalence of Type 2 Diabetes Mellitus (T2DM) (4). More than 42% of the population is overweight or obese (BMI ≥25 kg/m²), and around 6.28% is affected by T2DM (5, 6). From a women’s health perspective, obesity and T2DM are associated with adverse reproductive outcomes (7–11). In this context, science has sought to develop strategies to help patients with these conditions achieve healthier and longer lives. Recently, these strategies include the development of Glucagon-like peptide-1 receptor agonist (GLP-1RA) drugs. These drugs act by enhancing insulin secretion, suppressing glucagon release, delaying gastric emptying, and promoting satiety through acting directly in the hypothalamus (12). This drug-class, initially developed for the treatment of T2DM, was subsequently recognized as an effective weight loss treatment (13). Most GLP-1RA prescriptions remain within evidence-based clinical indications; however, off-label and cosmetic use is increasing, exposing more people to these drugs without medical counseling or monitoring. This is especially concerning for women of reproductive age, given the lack of safety evidence on GLP-1RA use during the perigestational period.

This growing off-label use is driven in large part by the pursuit of extreme beauty standards. The aesthetic pressure is even higher in younger women, with extreme thinness being increasingly associated with health and beauty, which is further promoted throughout social media, with negative outcomes including eating disorders (14). There is a particular concern regarding the cosmetic use of GLP-1RAs by women of reproductive age as well as by those using these medications for medical reasons. Considering that several of them are of childbearing age, it is expected that some of them may become pregnant during treatment, and there is a lack of scientific evidence regarding its safety during the perigestational period. Studies investigating the mechanisms and safety of GLP-1RAs are being published at an increasing pace; however, there are still limited data on animal studies, as well as human case reports, making it difficult to estimate its safety profile for human reproduction. Therefore, we aim to discuss current evidence on the potential impacts of GLP-1RA use during the perigestational period and to review the current clinical recommendations regarding their use in this period.

## GLP-1R agonists: mechanism and pharmacological development

Glucagon-like peptide-1 (GLP-1) is naturally synthesized in the L-cells of the intestine, pancreatic α-cells and the central neurons from the nucleus of the solitary tract (15). GLP-1 secretion increases after meals and is responsible for glucose homeostasis by binding to its receptor (GLP-1R); GLP-1RAs mimic the endogenous GLP-1 and activates GLP-1R (12). The main difference between endogenous GLP-1 and GLP-1RAs is related to their pharmacokinetic properties. GLP-1RAs have a prolonged half-life, allowing sustained receptor activation, and reduction in blood glucose concentrations in both fasting and postprandial periods (16). Currently available GLP-1RAs include exenatide, dulaglutide, liraglutide, and semaglutide, all administered subcutaneously, except for semaglutide, which also has an oral formulation. More recently, tirzepatide, a dual GLP-1/glucose-dependent insulinotropic polypeptide (GIP) receptor agonist, has also been approved. Only liraglutide, semaglutide and tirzepatide have been approved for weight loss.

Research recently focused on two main factors: structural similarity of the compounds to the endogenous GLP-1, and a longer half-life. Together, these characteristics would contribute to improving the therapeutic outcomes, as well as greater adherence to treatment due to the reduction in injection frequency (17). This is the main advantage of newer GLP-1RA compounds. For instance, semaglutide has an amino acid substitution that prevents dipeptidyl peptidase-4 degradation and the addition of a C18 fatty di-acid chain, even having 94% similarity to the endogenous GLP-1, it has a much longer half-life (from 144 to 168 hours) (18, 19).

## Reproductive implications and clinical considerations

Although GLP-1RAs were initially developed for the treatment of T2DM, the observation of significant weight loss in patients undergoing treatment sparked interest in the off-label use of these drugs for the treatment of obesity and other health conditions affected by glycemic imbalance, such as polyendocrine metabolic ovarian syndrome (PMOS) (20). Subsequently, some of these compounds were reformulated at different dosages, which are now marketed specifically for weight loss. This rapid increase gained visibility on social media platforms, reaching the lay public, popularizing discussions such as the phenomenon dubbed “Ozempic babies”, which is related to a possible baby boom association with GLP-1RAs use. However, scientific evidence does not support an increase in fertility or pregnancy rates associated with these medications (21).

Some unexpected pregnancies can be explained by the restoration of ovulation in individuals with obesity-related anovulation, as weight loss may lead to the resumption and regulation of ovulatory cycles (22). To minimize the risk of unplanned pregnancies and gestational exposure to these drugs, the use of long-acting reversible contraceptive methods, such as hormonal implants and intra-uterine devices is recommended for patients of childbearing age who use GLP-1Ras (22, 23). Contraceptive absorption does not appear to be affected by semaglutide and liraglutide, which may assist in reproductive planning and pregnancy timing (22). However, tirzepatide differs pharmacologically: due to its more pronounced effect on gastric emptying, it may influence the absorption of oral contraceptives (24, 25). In such cases, the use of a complementary barrier contraceptive method is recommended to prevent pregnancy (26). From a reproductive point of view, the long half-life of these drugs results in a prolonged window of potential embryonic exposure. There is also limited data on placental permeability of GLP-1RAs in humans and animal models. Therefore, women planning pregnancy are advised to undergo an 8-week washout period to avoid embryonic exposure to these drugs (22, 27). This recommendation is primarily based on the prolonged elimination half-life of semaglutide, which requires several weeks required for substantial drug clearance before conception (22, 27). While FDA and EMA recommendations are primarily based on product labeling and emphasize discontinuation before pregnancy, the 2025 joint clinical practice guideline from the Endocrine Society and the European Society of Endocrinology, similarly recommends discontinuation before conception while incorporating this recommendation into broader preconception care for individuals with T2DM (28). However, it is important to mention that, even if there is already a preconception guideline, current recommendations are insufficient to prevent accidental *in utero* exposure to these drugs. Evidence regarding the safety of GLP-1RA use during this critical developmental window remains limited. Therefore, these risks should be strongly highlighted during consultations with women of reproductive age. Regulatory agencies should also consider strategies to better inform patients about potential risks, including public awareness campaigns and updates to drug labelling.

## Real-world use and emerging concerns

Although most GLP-1RA prescriptions worldwide remain evidence-based for obesity and T2DM treatment, the use of these drugs is increasingly extending beyond their intended clinical purpose. A growing number of people are seeking this treatment for aesthetic reasons, fueling a parallel market outside regulated pharmaceutical distribution. In several countries, these drugs can only be obtained with a prescription; however, this parallel market includes unauthorized websites that sell them without a prescription or medical supervision, making it difficult to control and monitor their use (22). Additionally, there is strong media influence and social pressure that led to increased and unnecessary exposure of healthy women of reproductive age to GLP-1RAs. The increased use is driven by beauty standards reinforced through social media. Factors such as social media interactions, image sharing, gender, and sociocultural context, as well as time spent on social media, have all been associated with eating disorders (14). There has been a general increase in interest in GLP-1RAs formulations likely due to the dissemination carried out on social media, celebrities and influencers, as well as the extensive media coverage of the rapid weight loss effects of the medications (29). However, these medications were formulated to be used as a continuous treatment until patients’ weight is controlled, requiring medical follow-up to monitor weight loss and other health parameters. As a concern, GLP-1RAs use has been associated with significant adverse effects, such as loss of muscle mass, which implies the need for a healthy lifestyle, with a balanced diet and regular exercise (30).

## Evidence on gestational exposure: from experimental models to human data

Concerns regarding prolonged exposure during pregnancy, particularly during embryonic development, are justified, since experimental findings in animal models have already indicated semaglutide can induce congenital anomalies (31, 32).

FDA and the European Medicines Agency (EMA) pharmacological reviews reported studies of semaglutide conducted in rats, rabbits, and monkeys. In rats exposed from two weeks before mating until gestational day 17 (corresponding roughly to the first trimester) reduced growth, visceral anomalies in the heart and blood vessels, and skeletal anomalies in the cranial bones, vertebrae, and ribs were observed; males were exposed four weeks prior to mating (31, 33). The report mentions three doses similar to the MRHD, but it is unclear whether malformations vary among doses. EMA reports similar findings, adding brain ventricle malformations and impairment of nutrient supply across the yolk sac, without reporting doses or exposure duration (32). In rabbits exposed throughout gestational days 6–19 (corresponding roughly to the first trimester), low doses reduced maternal weight gain and food intake, while higher doses additionally caused early pregnancy losses and fetal abnormalities in the kidney, liver, and skeleton (31–33). In monkeys exposed between gestational days 16 and 50 (corresponding to late organogenesis and beyond), a dose-response effect was observed, with higher doses causing reduced maternal weight gain, maternal body weight loss of up to 16%, and increased pregnancy loss, as well as sporadic malformations in the vertebrae, sternum, and ribs (31–33). These findings occurred in the presence of maternal toxicity, including reduced food intake and substantial weight loss. This is an important consideration when interpreting the preclinical evidence, since maternal nutritional impairment may itself adversely affect embryofetal development, making it difficult to distinguish indirect effects related to maternal toxicity from direct drug-induced embryotoxicity. In addition, the reported developmental findings occurred across different exposure margins among animal species, which should also be considered when assessing the translational relevance of these findings.

Regarding human data, robust evidence supporting their safety during pregnancy remains lacking. A systematic review evaluated 1, 128 semaglutide-exposed pregnancies from five studies following gestation exposure to semaglutide (34). Negative outcomes, such as spontaneous abortion and preeclampsia, were associated with semaglutide in one study (34, 35); these findings are not consistent with other studies included in the review, which described spontaneous abortion rates similar to those observed in patients with diabetes and obesity (34, 36). When evaluating risk for malformations, the review found no significant increase in the risk of major congenital malformations with semaglutide use in pregnancy compared with insulin exposure (34, 37). However, the underlying maternal disease should be considered a potential confounder when interpreting comparisons with insulin-treated pregnancies, since diabetes itself is associated with adverse pregnancy outcome congenital malformations, macrosomia, and preeclampsia (38). Interestingly, the review reported a study in which treatment discontinuation was associated with fetal macrosomia and neonatal hypoglycemia, outcomes potentially related to T2DM itself (34, 39). Similarly, another study observed preterm birth and large-for-gestational-age infants, but no increased risk for congenital anomalies (34, 40). Notably, 80% of the semaglutide-exposed pregnancies in this cohort had a diabetes diagnosis, and the group also presented a substantially higher pre-pregnancy BMI than the unexposed population, suggesting that these adverse outcomes may largely reflect the underlying metabolic profile rather than a direct drug effect (40). Throughout the review, the authors emphasize the difficulty in evaluating these heterogeneous outcomes given the variability among the five studies reviewed, introducing significant bias into the conclusions. Semaglutide exposure does not appear to increase the risk of adverse gestational outcomes; however, the available evidence remains controversial and limited, highlighting important gaps regarding the safety of GLP-1RAs during pregnancy (34).

Another systematic review examined exposure to GLP-1 and dual GLP-1/GIP receptor agonists across the perigestational period (41). This review included 36 studies, which were grouped according to the type of exposure and study design. GLP-1RA drugs were not consistently associated with increased risk of major congenital anomalies or other adverse outcomes when large observational cohorts of periconceptional or first-trimester exposure were compared with insulin-treated or disease-matched controls. There was also considerable heterogeneity in maternal and obstetric outcomes, with no consistent pattern associated with drug use. The authors also highlighted the potential benefits of GLP-1RAs in the treatment of PMOS, as well as the lack of available data regarding lactation (41). These findings reinforce the need for further studies to investigate the mechanisms involved and establish causality.

Finally, a recent cohort study identified 529 pregnancies with periconceptional exposure to liraglutide or semaglutide (42). Propensity score matching was applied to compare women with periconceptional GLP-1RA exposure to unexposed women from the Danish National Prescription Register, matched by maternal and sociodemographic characteristics. Exposed pregnancies were stratified by maternal pre-existing diabetes status. Following the propensity score matching, only women with pre-existing diabetes exposed to GLP-1RA remained at increased risk of preterm birth, while prior to matching, exposed women exhibited higher rates of multiple obstetric complications. The authors conclude that premature birth rate observed in cases was related to diabetes-associated factors rather than the medication itself. However, there were considerable limitations on the cohort study that should be highlighted: the cohort was based on the Danish National Prescription Register, and the authors did not have access to information confirming exposure to the medication, which may lead to bias in the outcome’s confirmation (42).

This finding reflects a pattern that recurs throughout the literature: several adverse outcomes that appeared to be linked to GLP-1RA exposure, when adjusted for maternal diabetes and obesity, are more closely related to the underlying disease than to the drug itself. This reinforces the need for future studies to stratify by maternal diabetes and obesity status to more reliably assess GLP-1RA safety in pregnancy. Additionally, experimental animal study groups should also reflect this need, avoiding confounding factors caused by the metabolic changes inherent to diabetic or obese models. Underlying diseases directly impact reproductive outcomes, and that their treatment with GLP-1RA may reduce adverse reproductive outcomes by improving the underlying metabolic condition; however, most available human data are derived from women treated for obesity or diabetes, limiting direct extrapolation to healthy women using GLP-1RAs for cosmetic purposes. These considerations reinforce that the assessment of the drug’s direct effects on offspring must be evaluated and discussed responsibly in clinical practice and in the media, avoiding unnecessary embryofetal exposure.

In addition to the disease-related confounding discussed above, other methodological limitations of the available literature on GLP-1RA use during pregnancy should also be acknowledged. Many observational studies are subject to indication bias, since women with obesity or diabetes are more likely to be prescribed these drugs in the first place. Exposure misclassification may also occur, especially in studies based on prescription databases, since medication dispensation does not necessarily reflect actual use during pregnancy. Additionally, most available cohorts have relatively small sample sizes, which confer limited statistical power to detect rare congenital anomalies, whose expected frequency in the general population is already low. Together, these limitations reinforce that the absence of a consistent teratogenic signal to date reflects both the methodological limitations and the still insufficient volume of available data.

## Discussion and conclusion

In a society facing epidemics of obesity and T2DM, GLP-1RAs are increasingly becoming the first choice for patients with these conditions who need to lose weight. However, discussion of their risks and benefits remains necessary, especially regarding female reproductive health and pregnancy. Obesity and T2DM not only impair fertility but can also harm the developing embryo/fetus, making preconception weight management crucial. These conditions are themselves associated with adverse pregnancy outcomes, including preeclampsia, preterm birth, fetal macrosomia, and major congenital anomalies such as cardiac and neural tube defects. This creates additional challenges when distinguishing drug-related effects from teratogenic risks associated with pre-existing maternal conditions. We recognize the importance of the GLP-1RAs as a treatment for these conditions and their role in improving quality of life for patients in need. However, we also argue for responsible usage and mindful prescription, given the uncertainties that remain regarding their effects on future mothers and children.

The use of GLP-1RAs should be accompanied by strict contraceptive measures and a complete washout period under medical supervision is strongly recommended for patients planning pregnancy since experimental evidence suggests potential teratogenic effects (22, 27). The use of GLP-1RAs by healthy women of reproductive age for cosmetic purposes raises ethical and clinical questions that deserve attention through public health campaigns and clinical consultations that respect patient autonomy without compromising informed, evidence-based counseling. Despite the teratogenic findings in experimental models, human data are limited, unclear and inconclusive, highlighting important gaps in current literature. In addition to concerns regarding significant and rapid maternal weight loss during pregnancy, malnutrition is also a known teratogenic factor, raising questions about the underlying causes of the visceral malformations observed in the experimental models. The limited and inconsistent human data on teratogenicity is not equivalent to evidence of safety, and this distinction must guide clinical decision making and preconception counseling.

Monitoring and long-term follow-up babies born after GLP-1RAs exposure are essential, given the possibility of currently unidentified congenital anomalies. The main limitation highlighted in review studies is the lack of epidemiological studies. Therefore, further experimental and clinical studies investigating the mechanisms involved in the reproductive adverse effects should be prioritized and independently funded, reducing potential conflict of interest. Well-documented case reports are extremely valuable, even when no morphological abnormalities are observed. Given the popularity of these medications and their growing use among women of reproductive age, addressing these gaps through a broader and more translational research approach is urgently needed.
